# Internal Ophthalmoplegic Migraine During Pregnancy: A Clinical Case

**DOI:** 10.3390/neurolint16060128

**Published:** 2024-12-09

**Authors:** Brenda Castillo-Guerrero, Gloria Londoño-Juliao, Yesenia Pianetta, Melissa Gutiérrez-Rey, Bley Jair Zuñiga, Gustavo Pestana, Ana-Karina Carbonell-Zabaleta, Diego Rivera-Porras, Valmore Bermúdez, José Vargas-Manotas

**Affiliations:** 1 Universidad Simón Bolívar, Facultad de Ciencias de la Salud, Barranquilla 080001, Atlántico, Colombia; brenda.castillo@unisimon.edu.co (B.C.-G.); gloria.londono@unisimon.edu.co (G.L.-J.); yesenia.pianetta@unisimon.edu.co (Y.P.); melissa.gutierrez1@unisimon.edu.co (M.G.-R.); gustavopestanau@gmail.com (G.P.); ana.carbonell@unisimon.edu.co (A.-K.C.-Z.); jose.vargas@unisimon.edu.co (J.V.-M.); 2 Corpoclinic, Sincelejo 700001, Sucre, Colombia; reumatologia@corpoclinic.com.co; 3 Departamento de Productividad e Innovación, Universidad de la Costa, Barranquilla 080001, Atlántico, Colombia; drivera23@cuc.edu.co; 4 Clínica la Misericordia Internacional, Barranquilla 080001, Colombia; 5 Universidad Simón Bolívar, Facultad de Ciencias de la Salud, Centro de Investigaciones en Ciencias de la Vida, Barranquilla 080001, Colombia

**Keywords:** ophthalmoplegic migraine, pregnancy, cephalalgia, persistent mydriasis, pain, central nervous system

## Abstract

Background: Ophthalmoplegic migraine (OM) is an uncommon variant of migraine characterised by headache and cranial nerve palsy, posing significant diagnostic and therapeutic challenges. Objective: This study aimed to describe an extremely rare OM variant with a partial therapeutic response. Clinical Case: A 34-year-old pregnant woman in gestational week 19.1 (G6P2A3) with a history of three consecutive spontaneous abortions presented at the emergency services with insidious onset and mild-to-moderate-intensity pulsatile bifrontal headache for 15 days, and the positional changes exacerbated this. At peak intensity, she experienced nausea, vomiting, tinnitus, and photophobia without phonophobia or osmophobia, prompting multiple visits to the emergency department. Despite a broad range of treatments, including intravenous fluids, analgesia, pericranial blocks, and preventive management, there was a non-significative improvement in the symptomatology described above. However, spontaneous resolution of this clinical picture was observed during the postpartum period. Results: This case highlights the complexity of ophthalmoplegic migraine, especially in the context of pregnancy, and raises questions about the underlying pathophysiological mechanisms. The absence of structural lesions on neuroimaging and postpartum resolution suggests a potential association with the hormonal and physiological changes associated with pregnancy. Conclusions: Despite limited scientific evidence, this report contributes to expanding the knowledge of this rare entity and emphasises the importance of a multidisciplinary approach to its management.

## 1. Introduction

Migraine is a neurological disorder characterised by recurrent attacks of debilitating headaches accompanied by sensory and limbic symptoms in genetically predisposed individuals [1]. The clinical manifestations of migraine exhibit significant interindividual variability and even within the same individual, often posing diagnostic and therapeutic challenges due to the intricate network of brain circuits involved in its pathogenesis.

Migraine has been shown to have a significant impact on global health, as evidenced by the global burden of disease (GBD) study, a collaborative effort involving hundreds of researchers from around the world to quantify health loss from all causes of death and disability in all countries and regions, identifying migraine as the second leading cause of disability in all age groups and the leading cause among young people, with an estimated global preva-lence of 14% [2].

While most headaches (95%) present with typical symptoms, such as migraine and tension-type headaches [3], specific situations like pregnancy can induce changes in the phenomenology of primary migraines, manifesting as a fluctuation between forms with and without aura. Migraine without aura can appear in up to 10% of pregnancies, although several studies suggest even higher rates. Pre-existing migraines may sometimes intensify, especially during the early stages of pregnancy. On the other hand, migraines with aura show a greater susceptibility to begin or worsen during pregnancy, with onset rates exceeding 10–14% in some cases. Worsening affects 8% of women with migraines with aura [4]. Recent research has shown that hormonal changes during pregnancy can influence headache patterns, leading to the onset or exacerbation of primary headaches, with tension-type headaches occurring more frequently in women [5].

A small subset of migraine patients exhibit uncommon characteristics, posing diagnostic challenges. Migraine, in particular, can manifest in a variety of atypical and debilitating clinical presentations. Differentiating these migraines from other neurological conditions can, therefore, be challenging, potentially leading to delayed or incorrect treatment and even masking severe underlying diseases. Healthcare providers must remain vigilant towards recognising atypical migraine presentations to ensure optimal patient care and improve health outcomes [3].

Ophthalmoplegic migraine (OM) is an uncommon and poorly understood migraine variant. As a result, its definition and classification have been a subject of debate within the neuroscience community. In the first edition of the International Classification of Headache Disorders (ICHD) in 1988, OM was classified as a subtype of migraine. However, in the most recent edition, OM was reclassified as recurrent painful ophthalmoplegia (RPON). This reclassification was driven by the findings in nuclear magnetic resonance (NMR) studies exhibiting increased contrast enhancement and/or third cranial nerve thickening, suggesting neuropathy [4,5]. In this vein, in an attempt to differentiate patients with neuroimaging findings from those without, Friedman proposed a subdivision of RPON into distinct entities. He suggested that the absence of nerve enhancement or thickening indicates ophthalmoplegic migraine, a proposal that has not been universally accepted; thus, the aetiology and pathophysiology of this condition remain subjects of academic discussion [6].

Given the rarity of this condition and the fact that diagnosis is made only after excluding other potential causes, reporting such cases can improve our understanding of the pathology, clinical features, diagnosis, and treatment. For this reason, we present a case report of a variant of OM/RPON with bilateral internal ophthalmoplegia in a pregnant woman that resolved spontaneously after delivery.

## 2. Case Description

A 34-year-old woman with a history of three consecutive spontaneous abortions before the 10th week of gestation presented in May 2022, during gestational week 19.1 (G6P2A3), with a 15-day insidious onset, bifrontal, pulsatile headache, initially characterised by mild-to-moderate intensity, being exacerbated by positional changes. At peak intensity, she experienced nausea, vomiting, tinnitus, and photophobia without phonophobia or osmophobia, prompting multiple visits to the emergency department. During the last consultation, arterial hypertension was documented, leading to a diagnosis of chronic hypertension in pregnancy. As a result, alpha-methyldopa (250 mg orally twice daily) was initiated, with a subsequent reduction in the frequency and intensity of the headaches.

Two months after symptom onset, she experienced a new episode of headache, which progressed from mild-to-severe intensity over 6 h. Concurrently, she suffered a transient loss of consciousness while standing in a hot environment, accompanied by a 5 s episode of binocular vision loss. Immediately following the syncopal episode, she presented to the emergency department, where she received analgesics. She continued to experience blurred vision, which resolved spontaneously within 4 h. Considering the patient’s obstetric history and laboratory findings during her pregnancy follow-up at 25 weeks (positive lupus anticoagulant presumptive test and ANA positivity with a 1:160 titer) (Table 1), the possibility of antiphospholipid syndrome was raised. Consequently, specialised outpatient follow-up with perinatology was decided.

**Figure 1:**
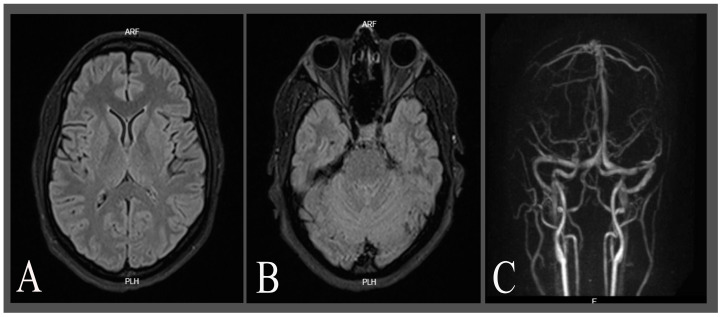
Non-enhanced brain MRI and time-of-flight magnetic resonance angiography (TOF-MRA). (**A**,**B**) show fluid attenuating recovery time (FLAIR) sequences showing intact grey and white matter structures with no signs of intracranial hypertension, evidence of lesions, haemorrhage, or other pathological abnormalities, and diffusion sequences. (**C**) shows a 3D reconstruction of a normal TOF-MRA.

One week later, she visited the emergency department again with a 3-day history of headaches with similar characteristics to previous episodes. On physical examination, the vital signs were within the normal limits. Fundoscopic examination, visual acuity, Ishihara, and Amsler grid tests were normal. Brain magnetic resonance imaging (MRI) and magnetic resonance angiography (MRA) with time-of-flight venous and arterial phases revealed no evidence of intracranial hypertension, T2 hyperintensities, vasculitis, or thrombosis (Figure 1). A subsequent lumbar puncture revealed an opening pressure of 33 cm H_2_O (NR: 6–25 cm H_2_O), with cerebrospinal fluid analysis showing no pathological findings and no pain improvement following the lumbar puncture. During hospitalisation, cephalalgia was resolved, but mydriasis persisted despite analgesic therapy, hydration, and pericranial block. The patient was discharged with a multidisciplinary follow-up plan involving neuro-ophthalmology, rheumatology, and neurology. Given the chronic and disabling nature of migraine in this patient, as well as the scarcity of medications evaluated during pregnancy and lactation, and the limitations of conventional prophylactic therapies in certain trimesters [7], the decision to initiate treatment was based on an individualised risk–benefit assessment. In this case, a combined therapeutic approach was chosen, including oral memantine at a dose of 10 mg daily and pericranial nerve blocks every 15 days. Additionally, enoxaparin 60 mg subcutaneously once daily, acetylsalicylic acid 100 mg daily, and prednisolone 10 mg orally daily were added until the end of the pregnancy.

During the outpatient follow-up in September 2022, the autoimmune profile was repeated. Although ANA remained positive at a 1:160 titer, the confirmatory lupus anticoagulant test, complementary laboratories, and other autoimmune markers were reported as negative (Table 1 and Table 2).

After ruling out neoplastic, vascular, and infectious etiologies and considering the presence of suggestive clinical manifestations, the diagnosis of seronegative obstetric antiphospholipid syndrome was proposed. A two-year follow-up with serial testing every 12 weeks confirmed the negativity of the autoantibodies (Table 1).

**Figure 2:**
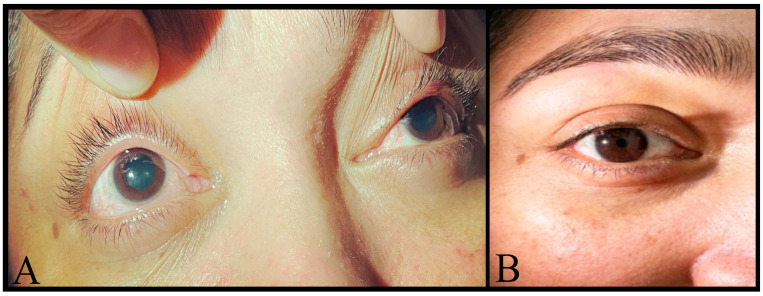
(**A**) Bilateral pupillary dilation with no photo motor or consensual response during hospitalisation; (**B**) reactive pupil to light and accommodation reflex after delivery.

A negative pilocarpine test ruled out a diagnosis of Adie’s tonic pupil. The intensity of migraines partially decreased with pericranial blocks; however, headache intensity increased in cycles of about 20 days, necessitating repeated medication with mydriasis persistence throughout the pregnancy. Because of that, at week 37th, lung maturity was induced, and a caesarean section was made without immediate complications. Remarkably, both migraine and mydriasis disappeared spontaneously on the 5th postpartum day, and she has remained asymptomatic since then (Figure 2).

## 3. Discussion

Ophthalmoplegic migraine was first described in 1860 by Gluber, who reported recurrent episodes of paresis and thickening of the third cranial nerve associated with headaches characteristic of migraine secondary to neurosyphilis. A second definition was provided in 1899 by the French neurologist Jules Déjerine, who described a patient suffering from unilateral headache accompanied by paralysis of the third cranial nerve. Déjerine termed this condition ‘migraine ophthalmique avec paralysie du moteur oculaire commun’. Möbius initially termed this condition as ‘periodic ocular paralysis’, while Charcot used the term ‘ophthalmoplegic migraine’ to describe a series of cases with this clinical presentation. Subsequently, in 1939, the American neurologist Harold Wolff described a series of cases with this type of headache and first proposed the term ‘ophthalmoplegic migraine’ to refer to this condition. Numerous cases of ophthalmoplegic migraine have since been published, allowing for a better understanding of its clinical characteristics, evolution, and therapeutic approaches [8].

The incidence of MO/RPON is approximately 0.7 cases per million inhabitants, classifying it as a rare disease. It commonly presents in children under the age of 10 and is diagnosed after at least two migraine attacks with paralysis of one or more cranial nerves (III, IV, or VI) in the absence of demonstrable intracranial lesions [9]. The patient, in this case, presented with an extremely rare variant known as bilateral internal ophthalmoplegic migraine, characterised by mydriasis associated with defects in visual accommodation and episodes of migraine headache. The term ‘internal’ refers to the absence of the paralysis of the extraocular muscles; thus, the patient did not exhibit ptosis, diplopia, or divergent strabismus. Neuroimaging did not reveal any contrast enhancement or thickening of the optic nerve. The most unique aspect of this case was the timing of its onset and resolution. The symptoms began during pregnancy and spontaneously resolved shortly after childbirth, which is an extremely rare occurrence in ophthalmoplegic migraine. To our knowledge, no similar case has been previously reported in the medical literature.

Several classic hypotheses have been proposed to explain the cause of cephalalgia in this context, such as thrombotic and platelet alterations [10]. Walsh and O’Doherty’s classic hypothesis of 1960 posited that inflammation of the internal carotid artery within the confines of the cavernous sinus, compressing the parasympathetic fibres on the surface of the third cranial nerve, could account for the pupillary dysfunction observed in this patient [7]. Sorsby-Vargas et al. suggested that trigeminovascular system activation during a migraine attack triggers the release of neuropeptides from the ophthalmic branch of the trigeminal nerve, inducing aseptic inflammation of intracranial vessels of various sizes, including the *vasa nervorum* and capillaries. Subsequent oedema leads to reflex vasoconstriction, nerve injury, and ischemia [9].

**Figure 3:**
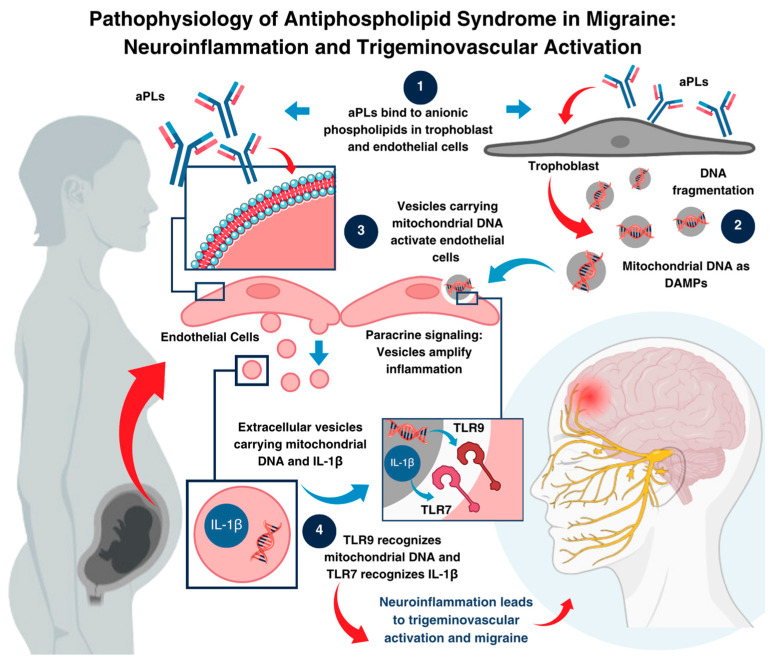
Pathophysiological mechanism of antiphospholipid syndrome Leading to migraine activation. This figure illustrates the hypothesised cascade involved in triggering migraine through four key steps. (**1**) Antiphospholipid antibodies (aPLs) bind to anionic phospholipids on the trophoblast and endothelial cell membranes, initiating the cascade. (**2**) This interaction results in mitochondrial DNA fragmentation within the trophoblast and the release of extracellular vesicles containing damage-associated molecular patterns (DAMPs), including mitochondrial DNA and pro-inflammatory cytokines such as interleukin 1 beta (IL-1β). (**3**) These vesicles are internalised by endothelial cells, where their contents are processed in endosomes. (**4**) Within the endosomes, TLR9 recognises mitochondrial DNA and TLR7 detects IL-1β, triggering a pro-inflammatory signalling cascade. This cascade sensitises the trigeminovascular system, initiating the mechanisms underlying migraine activation.

Nevertheless, considering the patient’s obstetric history, we propose that a seronegative antiphospholipid syndrome may have triggered an autoimmune process, leading to neuroinflammation and the subsequent development of MO/RPON. This hypothesis is supported by the extensive literature documenting a wide range of neurological manifestations in patients with aPL syndrome [11], such as transverse myelitis [12], stroke [13,14], TIA [13,14], seizures [15], cognitive dysfunction [16], chorea [17], multiple sclerosis [18], ocular symptoms [18], peripheral neuropathy [19], and migraine-like headaches [20,21,22]. The following figure (Figure 3) schematically illustrates the pathophysiological mechanisms of antiphospholipid syndrome in triggering migraine by activating the trigeminovascular system. It highlights the cascade initiated by the binding of antiphospholipid antibodies (aPLs) to anionic phospholipids, the subsequent release of extracellular vesicles, and the activation of Toll-like receptors (TLR7 and TLR9), leading to neuroinflammation and migraine development.

While our patient’s antinuclear antibodies were positive (1/160) with a persistent AC-18 pattern, it is important to note that up to 40% of the healthy population may exhibit these antibodies [23]. Moreover, in patients with other conditions, such as systemic sclerosis (SSc), this proportion can reach 40–50%, which might suggest an increased likelihood of lupus erythematosus (LE) as a secondary pathology [24]. However, it is crucial to consider that 5–7% of the population may present with an autoimmune disease, although systemic lupus erythematosus is relatively rare, with a prevalence not exceeding 0.1%. This high false-positive rate limits the specificity of ANA for the diagnosis of connective tissue diseases [23]. Therefore, despite this result, we cannot establish a definitive diagnosis for our patient, as she does not present clinical features that meet the classification criteria for any particular disease [24]. In rheumatic diseases, autoantibodies can appear years or even decades before disease development, and definitive diagnosis is clinical; thus, rheumatologic follow-up is strongly recommended [25].

Regarding pain management, given our patient’s poor response to acute and preventive therapies such as NSAIDs, acetaminophen, antiemetics, and beta-blockers, pericranial nerve blocks were initiated every 15 days, achieving partial pain control during this period. The choice of pericranial nerve blocks was based on their safety during pregnancy and the fact that their peripheral location results in considerably lower systemic effects than current medications [4]. Another preventive therapy option was the initiation of memantine, an N-Methyl D-Aspartate (NMDA) receptor antagonist, which has been explored as a preventive treatment for migraines due to its potential to modulate glutamatergic transmission, which is implicated in migraine pathophysiology [26]. Despite warnings about its use during pregnancy, including the lack of human studies and the potential risk of mild maternal toxicity at maximum doses [27], it was considered a valid therapeutic option due to its demonstrated efficacy, favourable tolerability, rapid titration time, and potentially more benign safety profile compared to other anti-migraine medications [28]. Although in our patient memantine did not completely resolve pain during her migraine attacks, its combination with peripheral nerve blocks provided some relief, especially at the beginning of its use.

Since MO/RPON diagnosis requires the exclusion of all other causes of cephalalgia and ophthalmoplegia, idiopathic intracranial hypertension was ruled out considering that, although our patient presented with migraine-like headaches that overlap with the migraine phenotype of this condition, she did not exhibit other less frequent but still characteristic symptoms such as kinetic sensation of the environment, cognitive alterations, radicular pain, or horizontal diplopia [29]. Additionally, there was an absence of radiological findings and persistence of headache following lumbar puncture. The elevated opening pressure observed in our patient can be attributed to increased intra-abdominal pressure caused by the gravid uterus, resulting in compression of the inferior vena cava and subsequent venous obstruction, leading to a redistribution of blood flow through the ascending lumbar vein and spinal venous plexus, ultimately decreasing cerebrospinal fluid volume and increasing epidural pressure [30].

Furthermore, NMR studies failed to reveal any space-occupying lesions within the cavernous sinus or orbit, nor were there any radiological signs of vasculitis, thrombosis, or idiopathic intracranial hypertension. Aneurysms were excluded as a potential cause. Tolosa–Hunt syndrome was considered unlikely due to the absence of the characteristic extraocular palsy and unilateral ocular pain. Sympathetic hyperreactivity was deemed an unlikely diagnosis given the lack of systemic symptoms such as tachycardia, diaphoresis, and hyperthermia. Finally, benign pupillary mydriasis was ruled out based on the absence of a pupillary light reflex.

## 4. Conclusions

Internal ophthalmoplegic migraine is an uncommon variant of migraine with an unclear and universally accepted pathophysiology. Given its extremely low incidence, evidence-based therapeutic protocols are lacking. Nevertheless, as seen in other headache types, a neuroinflammatory component, possibly autoimmune or triggered by physical, biological, or genetic stimuli, should be considered. Understanding this underlying mechanism is crucial for developing effective short- and long-term therapeutic strategies. To our knowledge, this is the first reported case of ophthalmoplegic migraine initiated during pregnancy and spontaneously resolving on the fifth postpartum day, with no subsequent recurrence. This case report highlights the importance of exhibiting the clinical course of a rare disease to examine the clinical response to pharmacological and non-pharmacological approaches in cephalalgia management.

## Figures and Tables

**Table 1:** Main immune test findings.

Laboratory Test	June, 2022 (2nd Trimester)	September, 2022 (3rd Trimester)	February, 2024 (Follow-Up)	May, 2024 (Follow-Up)	Reference Values
Anti-Beta2 Glicoprotein IgG	--	--	0.19 U/mL	0.18 U/mL	Negative: <0.8 U/mL Positive: >1.2 U/mL Undetermined: 0.8–1.2 U/mL
Anti-Beta2 Glicoprotein IgM	--	--	0.47 U/mL	0.31 U/mL	
Russell Viper Venom Test (LA1-TVVRD)	56.4 s	34.6 s	55.9 s	34.8 s	<1.20
Control (Day)	32.4 s	33.1 s	--	--	--
Confirmatory Test with Phospholipids (LA2)	--	36.4 s	32.5 s	--	--
Silica Clotting Time (SCT)	--	--	39.7 s	SCTSC: 33.7 s, Ratio: 0.86	<1.16
LA Ratio (LA1/LA2)	--	0.9	0.89	0.94	>2: positive. 1.5–2: moderate 1.2–1.5: weak
Anti-Cardiolipin IgM	8.94 MPL U/mL	--	4.3 MPL U/mL	10.2 MPL U/mL	Negative: <12 MPL U/mL Positive: >18 MPL U/ml
Anti-Cardiolipin IgG	2.86 GPL U/mL	--	2.9 GPL U/mL	1.9 GPL U/mL	Negative: <12 GPL U/mL Positive: >18 GPL U/mL
ANA *	1/160 patron lysosomal	1/160 Discrete cytoplasmic granular pattern	1/160 Discrete cytoplasmic granular pattern	1/160 Discrete cytoplasmic granular pattern	Positive: >1/80 ACR-EULAR 2019 (LES)
COOMBS (Direct)	--	Negative	--	--	--
Anti-DNA Abs	--	Negative	--	--	Negative: <1:10 Positive: >1:10
C3	--	--	123 mg/dL	115 mg/dL	90.0–180.0 mg/dL
C4	--	--	17 mg/dL	16 mg/dL	10.0–40.0 mg/dL
RO/SSA antibodies	--	0.3 U/mL	--	0.2 U/mL	Negative <0.8 U/mL Positive >1.2 U/mL
Anti-LA/SSB antibodies	--	0.1 U/mL	--	0.0 U/mL	
SM antibody	--	0.6 U/mL	--	0.2 U/mL	
Anti-RNP antibodies	--	0.2 U/mL	--	0.3 U/mL	
ANCA C and P	--	--	Negative	Negative	--
Factor V	37%	--	--	--	50–150%

Abbreviations: s: seconds, ANA: antinuclear antibody, C3 and C4: complement component 3 and 4, RO/SSA: anti-Ro/Sjögren’s syndrome antigen A, ANCA C: anti-neutrophil cytoplasmic antibodies cytoplasmic pattern, ANCA P: anti-neutrophil cytoplasmic antibodies perinuclear pattern, Factor V: coagulation factor V. * *The antinuclear antibodies were interpreted with a cutoff of >1:160 to determine their clinical significance.*

**Table 2:** Complementary laboratory tests.

Test	Results at Week 25 (2022)	February, 2024 (Follow-Up)	May, 2024 (Follow-Up)	Reference Values
Haemoglobin	12.30 g/dL	13.9 g/dL	14.9 g/dL	11.20–15.70 g/dL
Platelets	260 ×10^3^/μL	203 ×10^3^/μL	244 ×10^3^/μL	140–400 ×10^3^/μL
Leucocytes count	10.04 ×10^3^/mm^3^	7.88 ×10^3^/mm^3^	7.59 ×10^3^/mm^3^	4.00–10.04 ×10^3^/mm^3^
Proteinuria	Negative	--	--	Negative <150 mg/day or <10 mg/dL Positive >10 mg/dL or >150 mg/day
Urinary leucocytes	0–3 cells/high-power field	--	--	0–5 cells/high-power field
Urinary red cells	0–2 cells/high-power field	--	--	0–5 cells/high-power field
24 h proteinuria	231.00 mg/24 h			10.00–140 mg/24 h

## Data Availability

Data sharing is not applicable to this article.
